# Supplementary material for: Intermittent Theta Burst Stimulation Combined with Cognitive Training to Improve Negative Symptoms and Cognitive Impairment in Schizophrenia: A Pilot Study
Source: Brain Sci. 2024 Jul 8;14(7):683. doi: 10.3390/brainsci14070683 (PMC11274516; doi:10.3390/brainsci14070683)
Supplement: Supplementary file 1 [file brainsci-14-00683-s001.zip › brainsci-3059178-supplementary.pdf]

Supplementary Materials "Intermittent theta burst stimulation combined with cognitive training to improve negative symptoms and cognitive impairment in schizophrenia: a pilot study"

Vergallito Alessandra, Gesi Camilla, Torriero Sara

Section S1 – Exercises details

We report here the Cogpack exercises used during our training sessions. Each type of exercise included different tasks, varying in difficulty, type of stimuli, and presentation modality.

MEMORY
REACTION
ROUTE
CONNECT
COMPARISONS
EYEWITNESS
SEQUENCE
MEMORY
SEARCH
LABYRINTHS
SCALES
MATH
CONFUSION
COLOR&LABELS

Training material in learning and memory tasks comprised visual and verbal stimuli, complex ecological scenarios, or visuospatial paths. Verbal stimuli could be presented visually, acoustically, or by combining the two modalities. The number of items presented, the request to recognize or recall the elements, and the scene complexity varied across the tasks. Exercises targeting speed processing typically required choosing as fast as possible whether a couple of stimuli were equal or different, with task complexity manipulated through an increasing number of features to check. Working memory training could include arithmetical calculations and tasks in which sequences (numerical, months, weekdays) were presented, and participants should respond (or not) according to specific rules (for example, they should press every time a number was one unit larger than the one presented immediately before).

Section S2 – Details on the statistical approach and model selection

The choice of the proper statistical approach was not easy due to the small sample size. We primarily considered non-parametric statistics and linear mixed models and carefully considered the advantages and limitations of the two approaches. On the one hand, non-parametric statistics are used with non-normal distributions. However, they are not allowed with less than five patients (as in some of our experimental conditions) [105], are influenced by missing data, and did not allow us to observe one of our primary interests, namely the simple effects of iTBS and CR, plus the combined interventions. On the other hand, linear mixed models allow us to account for individual differences, which are substantial in schizophrenic patients, are not influenced by missing data [102], and facilitate the analysis of the effects of stimulation and training over time and the interaction between the two in modulating cognitive impairment and negative symptoms, which is a crucial aim in the current study. Moreover, previous studies suggested the feasibility of applying linear mixed models in small sample size research and single-case studies compared to a control group [102, 106-107], but see [108]. Although non-optimal, considering the data distribution of our longitudinal study, we opted to apply linear mixed models in our analyses, even though we openly acknowledged sample size limitation by addressing it throughout the manuscript, highlighting the visual presentation of data trends, and releasing all the collected data on OSF so that other authors can use or check on them.

Model selection

The following tables summarize the model-simplification procedure for each measurement, including the goodness-of-fit tests and their results. The rightmost part of each table reports the effects of the included variables.

Table S1. Results of the mixed-effect analysis on negative symptoms comparing pre- vs. post-treatment scores.

Parameter	χ^2	***P***	***Removal order***	χ^2	***Df***	***p***
Time	-	-	*Not removed*	0.5165	1	.4723
Stimulation	-	-	*Not removed*	0.0566	1	.8120
*Time*Stimulation*	-	-	*Not removed*	3.5486	1	.0596
*Training*Stimulation*Time*	1.603	.2055	1	-	-	-
*Stimulation*Training*	1.6099	.2045	2	-	-	-
*Training*Time*	1.6802	.1949	3	-	-	-
Training	0.3586	.5493	4	-	-	-

Table S2. Results of the mixed-effect analysis on negative symptoms comparing all time points.

Parameter	χ^2	***P***	***Removal order***	χ^2	***Df***	***p***
*Training*Stimulation*Time*	5.1405	.2732	1	-	-	-
*Training*Time*	3.905	.4190	2	-	-	-
*Stimulation*Time*	6.0292	.1970	3	-	-	-
Time	5.9183	.2053	4	-	-	-
*Stimulation*Training*	2.9720	.0847	5	-	-	-
Stimulation	0.0063	.9369	6	-	-	-
Training	0.1038	.7473	7	-	-	-

The interaction between stimulation and training was explored since it showed a trend. However, the simple effects of Stimulation ($\chi^2_{(1)}$=0.01, p=.937) and Training ($\chi^2_{(1)}$=0.1, p=.749) and their interaction ($\chi^2_{(1)}$=2.6, p=.108) were not significant. For brevity, we reported in the main text that the best-fitting model did not include fixed factors.

Table S3. Results of the mixed-effect analysis on MCCB total scores comparing pre vs. post-treatment scores.

Parameter	χ^2	***P***	***Removal order***	χ^2	***Df***	***p***
Time	-	-	*Not removed*	11.1350	1	<.001
*Training*Stimulation*Time*	0.3585	.5493	1	-	-	-
*Stimulation*Time*	0.0001	.9914	2	-	-	-
*Training*Time*	0.3316	.5647	3	-	-	-
*Training*Stimulation*	1.6561	.1981	4	-	-	-
Stimulation	0.6513	.4196	5	-	-	-
Training	2.1681	.1409	6	-	-	-

Table S4. Results of the mixed-effect analysis on MCCB at the five time points.

Parameter	χ^2	***P***	***Removal order***	χ^2	***Df***	***p***
*Training*Stimulation*	-	-	*Not removed*	2.7457	1	.0975
Stimulation	-	-	*Not removed*	1.3876	1	.2388
Training	-	-	*Not removed*	2.8461	1	.0916
Time	-	-	*Not removed*	36.0480	4	<.001
*Training*Stimulation*Time*	3.9757	.4093	1	-	-	-
*Stimulation*Time*	3.067	.5467	2	-	-	-
*Training*Time*	6.2152	.1836	3	-	-	-

The interaction between stimulation and training, plus the simple effect of time, was explored since it was considered the best-fitting model. However, the simple effect of Stimulation ($\chi^2_{(1)}$=1.4, p=.239) and Training ($\chi^2_{(1)}$=2.7, p=.098) and their interaction ($\chi^2_{(1)}$=2.8 p=.092) were not significant. For brevity, we reported in the main text only the main effect of time.

Table S5. Results of the mixed-effect analysis on verbal learning comparing pre vs. post-treatment scores.

Parameter	χ^2	***P***	***Removal order***	χ^2	***Df***	***p***
Time	-	-	*Not removed*	3.2966	1	.0694
Training	-	-	*Not removed*	0.1918	1	.6614
*Time * Training*	-	-	*Not removed*	3.8136	1	.0508
*Training*Stimulation*Time*	0.0529	.8180	1	-	-	-
*Stimulation*Time*	0.0579	.8098	2	-	-	-
*Training*Stimulation*	0.4454	.5044	3	-	-	-
Stimulation	0.0131	.9089	4			

Table S6. Results of the mixed-effect analysis on verbal learning at the five time points.

Parameter	χ^2	***P***	***Removal order***	χ^2	***Df***	***p***
Time	-	-	*Not removed*	7.9888	4	.0920
Training	-	-	*Not removed*	0.5681	1	.4510
*Time * Training*	-	-	*Not removed*	7.8809	4	.0960
*Training*Stimulation*Time*	3.4038	.4927	1	-	-	-
*Stimulation*Training*	0.0145	.9042	2	-	-	-
*Stimulation*Time*	3.1455	.5338	3	-	-	-
Stimulation	0.1245	.7242	4	-	-	-

Table S7. Results of the mixed-effect analysis on visual learning comparing pre- vs. post-treatment scores.

Parameter	χ^2	***P***	***Removal order***	χ^2	***Df***	***p***
Time	-	-	*Not removed*	4.1945	1	.0406
Training	-	-	*Not removed*	5.5121	1	.0189
*Training*Stimulation*Time*	0.0225	.8807	1	-	-	-
*Stimulation*Training*	0.0866	.7686	2	-	-	-
*Stimulation*Time*	0.0581	.8096	3	-	-	-
Stimulation	0.0881	.7666	4	-	-	-
*Time*Training*	0.1721	.6783	5	-	-	-

Table S8. Results of the mixed-effect analysis on visual learning at the five time points.

Parameter	χ^2	***P***	***Removal order***	χ^2	***Df***	***p***
Training	-	-	*Not removed*	6.2684	4	.0123
Time	-	-	*Not removed*	8.1385	4	.0866
Stimulation	-	-	*Not removed*	1.1937	1	.2746
*Time*Stimulation*	-	-	*Not removed*	7.4524	4	.1138
*Training*Stimulation*Time*	6.7891	. 1475	1	-	-	-
*Time*Training*	0.4021	.9823	2	-	-	-
Stimulation Training*	0.3950	.5297	3	-	-	-

Table S9. Results of the mixed-effect analysis on speed processing comparing pre- vs. post-treatment scores

Parameter	χ^2	***P***	***Removal order***	χ^2	***Df***	***p***
Time	-	-	*Not removed*	4.6848	1	.0304
*Training*Stimulation*Time*	0.8445	.3581	1	-	-	-
*Stimulation*Time*	0.0348	.8520	2	-	-	-
*Training*Time*	0.1513	.6973	3	-	-	-
*Stimulation*Training*	0.9892	.3199	4	-	-	-
Stimulation	0.4782	.4892	5	-	-	-
Training	2.0169	.1556	6	-	-	-

Table S10. Results of the mixed-effect analysis on the speed processing at the five-time points.

Parameter	***χ2***	***P***	***Removal order***	***χ2***	***Df***	***p***
Time	-	-	*Not removed*	28.5950	4	< .001
*Training*Stimulation*Time*	3.0908	.5428	1	-	-	-
*Training*Time*	1.3905	.8458	2	-	-	-
*Stimulation*Time*	1.5535	.8171	3	-	-	-
*Stimulation*Training*	2.2182	.1364	4	-	-	-
Stimulation	0.2234	.6365	5	-	-	-
Training	2.4766	.1156	6	-	-	-

Table S11. Results of the mixed-effect analysis on vigilance comparing pre- vs. post-treatment scores.

Parameter	χ^2	***P***	***Removal order***	χ^2	***Df***	***p***
Stimulation	-	-	*Not removed*	0.5630	1	.4531
Training	-	-	*Not removed*	0.7272	1	.3938
*Stimulation*Training*	-	-	*Not removed*	3.7310	1	.0534
*Training*Stimulation*Time*	0.0079	.9291	1	-	-	-
*Training*Time*	0.4911	.4834	2	-	-	-
*Stimulation*Time*	0.5115	.4745	3	-	-	-
Time	1.5717	.2100	4	-	-	-

Table S12. Results of the mixed-effect analysis on vigilance at the five time points.

Parameter	χ^2	***P***	***Removal order***	χ^2	***Df***	***p***
Time			*Not removed*	17.4282	4	.0016
Stimulation	-	-	*Not removed*	0.8885	1	.3459
Training	-	-	*Not removed*	0.5665	1	.4517
*Stimulation*Training*	-	-	*Not removed*	5.8897	1	.0152
*Training*Time*	-	-	*Not removed*	8.5807	4	.0725
*Training*Stimulation*Time*	7.6698	.1045	1	-	-	-
*Stimulation*Time*	3.0715	.5459	2	-	-	-

Table S13. Results of the mixed-effect analysis on working memory comparing pre- vs. post-treatment scores.

Parameter	χ^2	***P***	***Removal order***	χ^2	***Df***	***p***
Time	-	-	*Not removed*	6.7719	1	.0093
Training	-	-	*Not removed*	0.0922	1	.7615
Stimulation	-	-	*Not removed*	2.5313	1	.1116
*Training*Stimulation*	-	-	*Not removed*	5.5023	1	.0190
*Training*Stimulation*Time*	0.0556	.8136	1	-	-	-
*Stimulation*Time*	0.0007	.9783	2	-	-	-
*Training*Time*	1.2784	.2582	3	-	-	-

Table S14. Results of the mixed-effect analysis on working memory comparing the five data points.

Parameter	χ^2	***P***	***Removal order***	χ^2	***Df***	***p***
Time			*Not removed*	16.4190	4	.0025
Training	-	-	*Not removed*	0.3315	1	.5648
Stimulation	-	-	*Not removed*	2.6500	1	.1035
*Training*Stimulation*			*Not removed*	4.1073	1	.0427
*Training*Stimulation*Time*	2.7404	.6022	1	-	-	-
*Stimulation*Time*	2.8612	.5813	2	-	-	-
*Training*Time*	5.5661	.2340	3	-	-	-

Table S15. Results of the mixed-effect analysis on problem-solving comparing pre- vs. post-treatment scores.

Parameter	χ^2	***P***	***Removal order***	χ^2	***Df***	***p***
Stimulation	-	-	*Not removed*	0.0046	1	.9461
Time	-	-	*Not removed*	0.9307	1	.3347
Training	-	-	*Not removed*	0.2894	1	. 5906
*Stimulation*Time*	-	-	*Not removed*	0.9895	1	.3199
*Training*Stimulation*	-	-	*Not removed*	0.7399	1	.3897
*Training*Time*	-	-	*Not removed*	0.0421	1	.8375
*Training*Stimulation*Time*	-	-	*Not removed*	3.5028	1	.0613

Table S16. Results of the mixed-effect analysis on problem-solving comparing the five data points

Parameter	χ^2	***P***	***Removal order***	χ^2	***Df***	***p***
Stimulation	-	-	*Not removed*	0.1957	1	.6582
Time	-	-	*Not removed*	11.2388	4	.0240
Training	-	-	*Not removed*	0.2145	1	.6432
*Stimulation*Time*	-	-	*Not removed*	4.872	4	.3007
*Training*Stimulation*	-	-	*Not removed*	0.5651	1	.4522
*Training*Time*	-	-	*Not removed*	1.3871	4	.8464
*Training*Stimulation*Time*	-	-	*Not removed*	7.2422	4	.1236

Section S3 – Supplementary results

The best-fitting model for visual learning included the simple effects of time ($\chi^2_{(1)}$ = 4.2, p = .041) and training ($\chi^2_{(1)}$ = 5.5, p = .019). Considering the time effects, scores were higher after the treatment than at baseline. Concerning the simple effect of training, participants assigned to the training condition had higher scores than those not receiving it. When including the follow-ups, the training effect remained significant ($\chi^2_{(1)}$ = 6.3, p = .012). The best-fitting model also included the interaction between time and stimulation. However, such interaction ($\chi^2_{(4)}$ = 7.5, p = .114) and the simple effects of time ($\chi^2_{(4)}$ = 8.1, p = .087) and stimulation ($\chi^2_{(1)}$ = 1.2, p = .275) were not significant (see Figure S1).

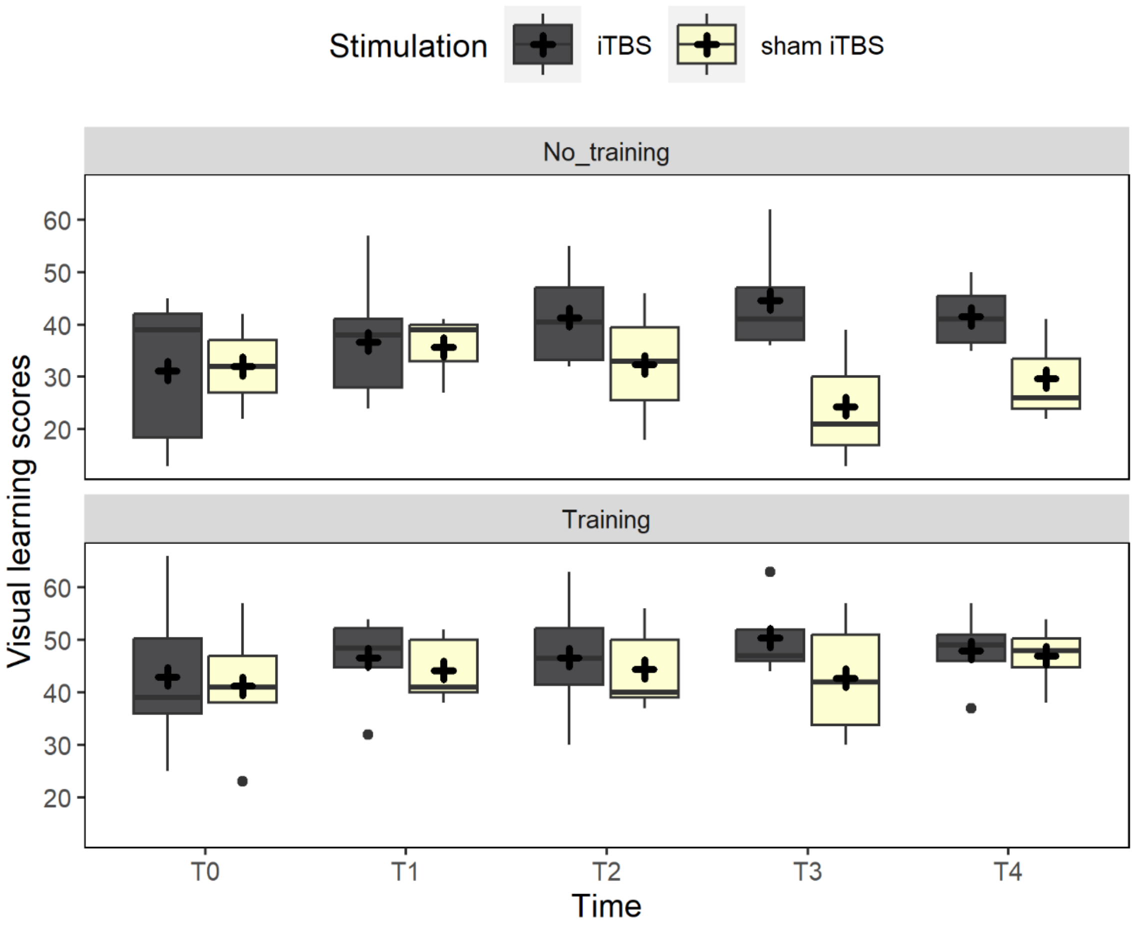

Figure S1. The figure depicts the scores for visual learning at the five different time points. The boxplots compare real iTBS (dark gray boxes) and sham iTBS (light yellow boxes) for the no training and training conditions. Black dots represent outliers, and the cross symbol represents the mean values.

The best-fitting model on speed processing included the effect of time ($\chi^2_{(1)} = 4.7$, $p = .030$): participants performed better after treatment than at baseline. The effect of time was maintained, including the follow-ups ($\chi2_{(4)} = 28.6$, $p < .001$), with improvements at T2, T3 ($p = .001$), and T4 ($p <.001$) compared to baseline and at T4 compared to T1 (p = 036) (see Figure S2).

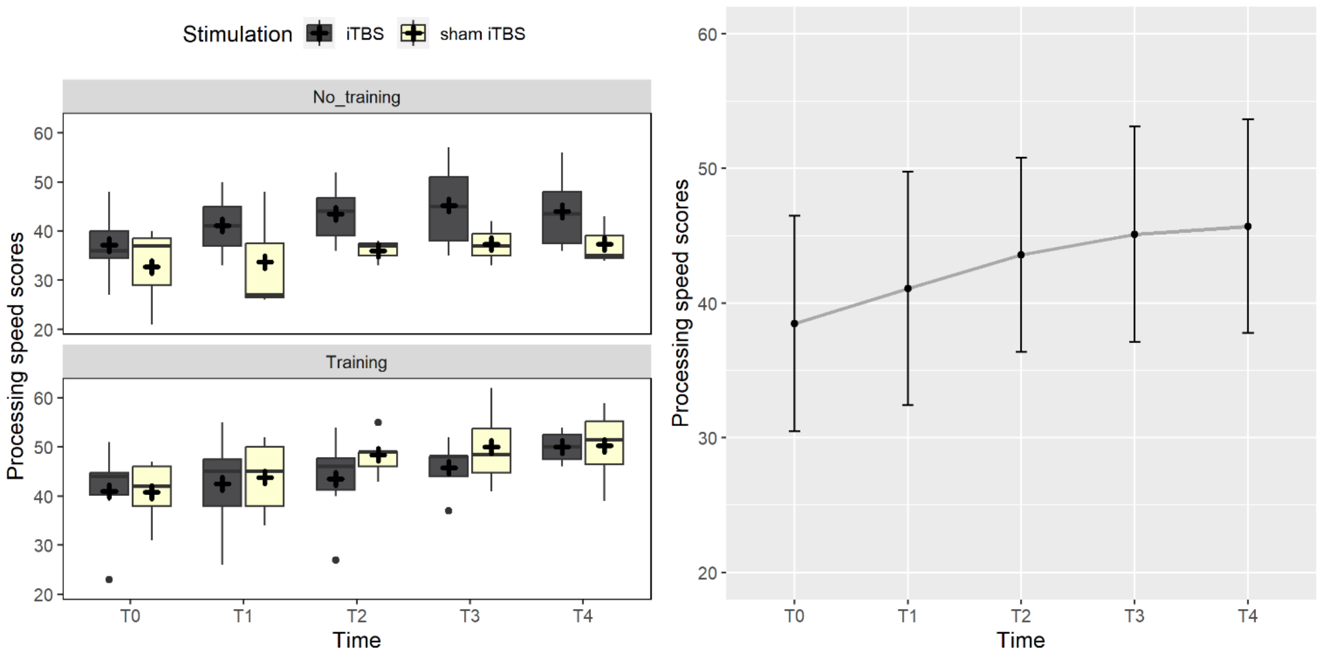

Figure S2. The figure represents the trend of speed of processing scores. On the left panel, the boxplots compare real iTBS (dark gray boxes) and sham iTBS (light yellow boxes) in the no training and training conditions. Black dots represent outliers, and the cross symbol represents the mean values. On the right panel, the simple effect of time is represented.

Considering the working memory performance, the best-fitting model included the interaction between training and stimulation ($\chi^2_{(1)} = 5.5$, $p =.019$) and the simple effect of time ($\chi^2_{(1)} = 6.8$, $p = .009$). Considering the interaction between training and stimulation, participants who did not receive the training had higher scores in the real than in the sham stimulations ($p = .010$). Conversely, performance for the two stimulations did not differ in the training condition ($p = .725$). The simple effect of time highlighted that participants improved at T1 compared to T0. When including the follow-up measures, the best-fitting model included the same parameters. The interaction between stimulation and training remained significant ($\chi^2_{(1)} = 4.1$, $p = .043$), maintaining the difference between participants not receiving the training in the real vs. sham conditions ($p = .019$). The simple effect of time ($\chi^2_{(4)} = 16.4$, $p = .003$) highlighted that scores were significantly higher at all time points compared to the baseline ($ps < .046$) (see Figure S3).

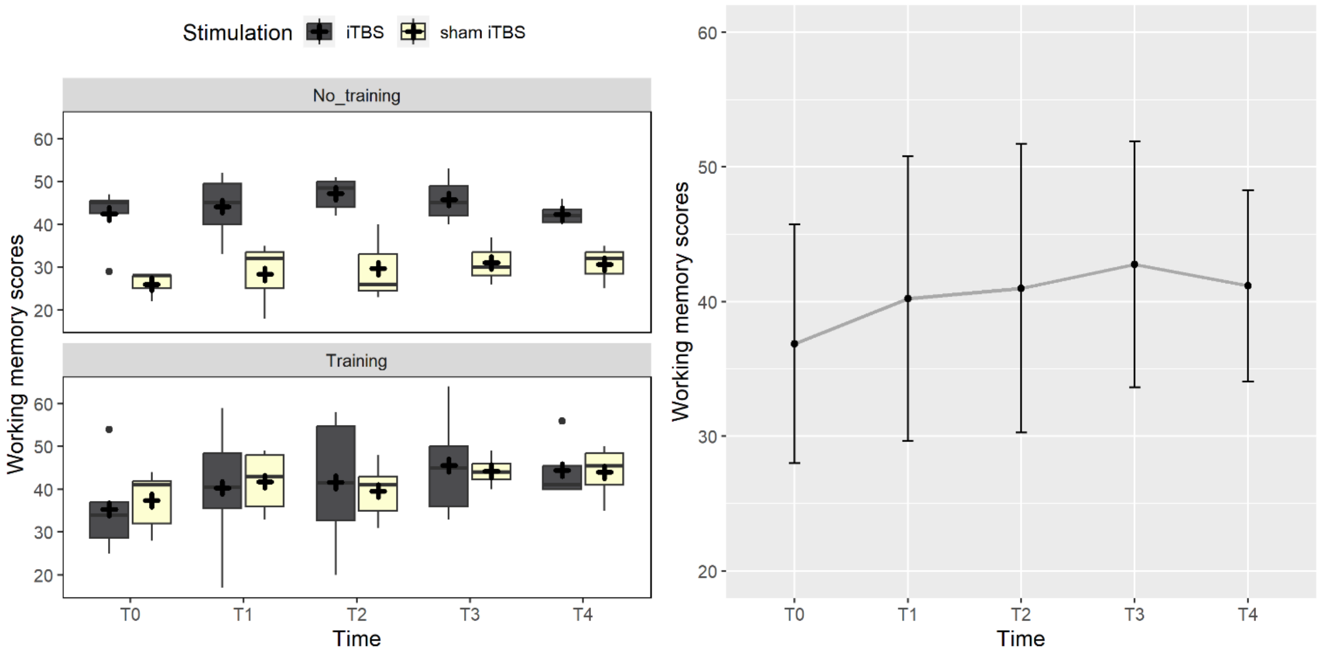

Figure S3. The figure represents the trend of working memory scores. The left panel depicts the working memory scores at the five different time points. The boxplots compare real iTBS (dark gray boxes) and sham iTBS (light yellow boxes) for the no training and training conditions. Black dots represent outliers, and the cross symbol represents the mean values. The right panel represents the simple effect of time.

For the problem-solving scores, the best-fitting model included the three-way interaction among time, stimulation, and training ($\chi^2_{(1)}$ = 3.5, p = .061). Post-hoc analyses did not reveal significant comparisons (all ps > .157). The full model was chosen as best-fitting also when adding all the data points, but only the main effect of time emerged as significant ($\chi^2_{(4)}$ = 11.2, p = .024), with a trend for higher scores at T3 compared to T0 (p = .069) (see Figure S4).

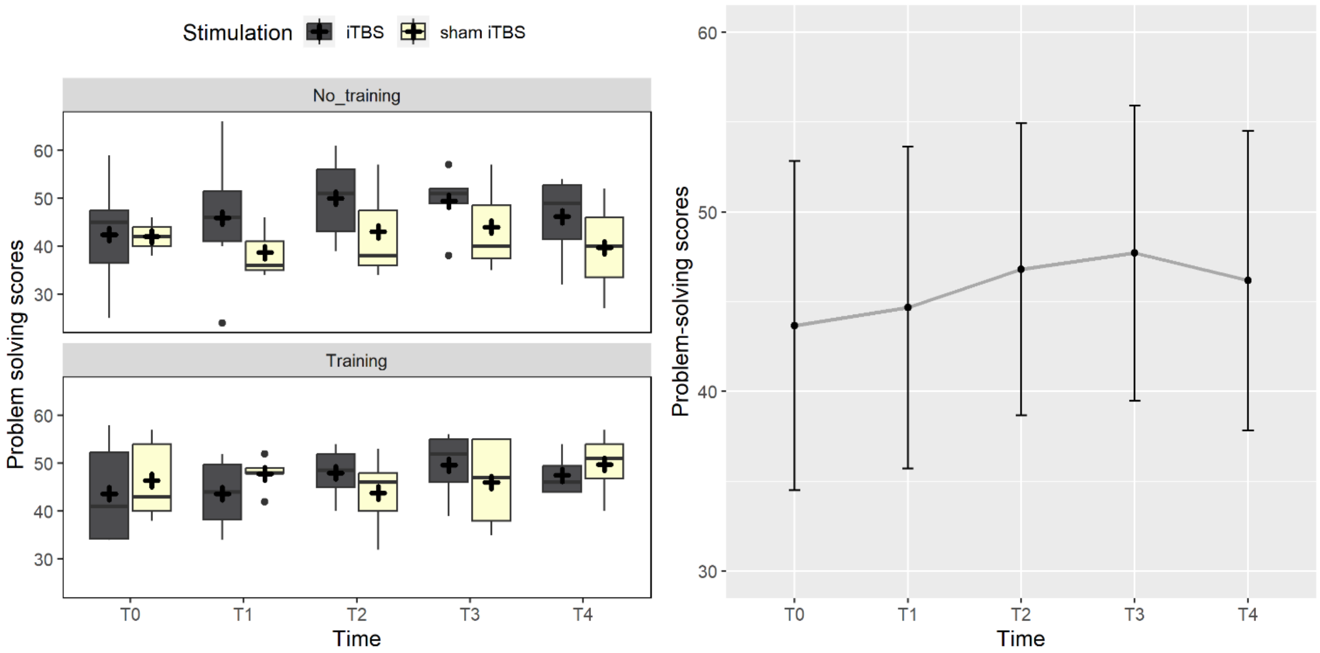

Figure S4. The figure represents the problem-solving scores trend. On the left panel, the boxplots compare real iTBS (dark gray boxes) and sham iTBS (light yellow boxes) in the no training and training conditions. Black dots represent outliers, and the cross symbol represents the mean values. On the right panel, the simple effect of time is represented.
